# Correlations of palatal surface area with anthropometric dimensions of the head and face

**DOI:** 10.1007/s00276-022-03008-4

**Published:** 2022-09-02

**Authors:** Z. Assy, D. H. J. Jager, H. S. Brand, F. J. Bikker

**Affiliations:** 1 Department of Oral Biochemistry, Academic Centre for Dentistry Amsterdam, University of Amsterdam and VU University Amsterdam, Room 12N-37, Gustav Mahlerlaan 3004, 1081 LA Amsterdam, The Netherlands; 2 Department of Maxillofacial Surgery and Oral Pathology, Amsterdam UMC and Academic Centre for Dentistry Amsterdam (ACTA), Vrije Universiteit Amsterdam, Amsterdam Institute for Infection and Immunity Amsterdam, Amsterdam, The Netherlands

**Keywords:** Anthropometric measurements, Palatal surface area, Head and face dimensions, TRIOS 3 scanner

## Abstract

**Purpose:**

Saliva distribution over the palatal surface plays an important role in the perception of dry mouth. It is envisaged that non-invasive estimation of the palatal surface area by anthropometric measurements of the head and face can be useful in the assessment of oral dryness. For this purpose, the relationship between the palatal surface area and anthropometric measurements of the head and face was investigated.

**Methods:**

The palatal surface was measured in 51 healthy volunteers using an intra-oral scanner. The distances between anthropometric landmarks of the head and face were determined using an anatomical sliding caliper. Correlations between the palatal surface area and the anthropometric landmarks were investigated.

**Results:**

The median palatal surface area for the total study population was found to be 2120.6 mm^2^. Virtually, all anthropometric measurements showed significant differences between females and males. Various head and face measurements had a significant correlation with the palatal surface area. However, these correlations disappeared when the participants were stratified based on their sex, with the exception of mandibular length and palatal width in females.

**Conclusion:**

The surface area of the palate correlates with nearly all anthropometric measurements of the head and face included in this study. Yet, the clinical applicability seems limited to females.

## Introduction

Human saliva is predominantly produced by three pairs of major glands known as parotid, submandibular, and sublingual glands. These glands are responsible for the production of 90% of the volume of saliva [8]. Each of the glands excretes saliva with a unique consistency into the oral cavity via various salivary ducts [14]. The openings of these salivary ducts are located in various intra-oral locations, such as the buccal mucosa for the parotid glands and the floor of the mouth for the sublingual and submandibular glands [14].

After secretion, saliva is distributed over the various intra-oral surfaces, especially during chewing and swallowing [16, 29]. Several studies have explored the thickness of the salivary film covering the teeth and oral mucosa at various intra-oral locations [6, 15, 28]. The salivary film thickness at the anterior part of the palate seems to be relatively thin compared to other intra-oral surfaces [4, 5, 10, 18, 22, 23, 25, 29, 30]. In addition, in patients suffering from hyposalivation, a reduced salivary film thickness at the anterior palate was observed compared to healthy controls [4, 5, 10, 18, 22, 23, 25, 29, 30].

Next to e.g. the salivary volume, the size of the surface area of the intra-oral regions relates to the salivary film thickness. To investigate the surface area of the oral cavity, previous studies used the so-called foil technique; stone models of dental impressions were prepared and covered with aluminium foil. Subsequently, this foil was weighed to deduce the surface area [6, 15, 28]. Despite the fact that this foil technique has been proven to be reproducible [6, 15, 28] some drawbacks were noted as well; adaptation of the foil onto the models without stretching appeared challenging. Besides, it was difficult to fold the foil completely into interdental spaces, and around the labial and buccal vestibular mucosa [6]. Therefore, in a recent study an alternative strategy was explored using cone-beam computed tomography (CBCT) in combination with digital analysis [3]. However, in contrast to the studies which used the foil technique, the CBCT analysis was performed on cadavers [3]. It was found that CBCT analysis had good reliability for measuring various intra-oral surface areas such as the palate, tongue, mucosa, and hard tissues. The studies using the foil technique and the CBCT analysis showed identical results for the palatal surface area (20.1 ± 1.9 vs. 20.0 ± 2.9 cm^2^) [3, 6]. In the cadaver study, the sizes of several intra-oral surface areas, including the palatal surface area, were related to facial anthropomorphic measurements [3]. Moderate, yet statistically significant correlations were observed between the palatal surface area and the length of the head, as well as the surface area of the tongue and the depth of the head [3].

However, it was postulated that the study was limited by the fact that soft tissues of the cadavers were solidified by their embalmment in a formaldehyde solution which would lead to a suboptimal approximation of the surface areas [3]. For this reason, in the current study, we included living subjects and also applied an intra-oral scanner, which projected a light source on the intra-oral surfaces to be scanned. Then, images captured by imaging sensors are processed by scanning software to produce triangulated point clouds that enable a virtual 3D surface model to be created [7]. A recent study revealed promising results using this scanner, especially for the documentation of palatal soft tissue in terms of shape, colour, and curvature [9]. Therefore, this study was designed to validate the relation between the palate surface area, measured using an intra-oral scanner, and anthropometric measurements of the head and face in living subjects. A relation between the anthropomorphic measurements and the palatal intra-oral surfaces would potentially enable easy approximation of the palatal intra-oral surface area in a chairside medical setting. Approximation of the palatal surface area might be relevant for clinicians investigating the oral cavity, such as dentists and oral maxillofacial surgeons.

## Material and methods

### Participants

The study was approved by the Ethics Review Committee at the Academic Centre for Dentistry Amsterdam (ACTA; 202065). Volunteers were recruited at ACTA through posters. Eligibility criteria required volunteers to be 18 years or older. Informed written consent was obtained from all volunteers. Data analysis of volunteers was completed anonymously, and only age and sex were registered. The reporting of this study conformed to the STrengthening the Reporting of OBservational studies in Epidemiology (STROBE) statement [27].

A priori sample size calculation was performed using G*Power software, version 3.1.9.4 (Heinrich-Heine-Universität Düsseldorf, Düsseldorf, Germany); the correlation coefficient of a previous study was used: 0.59 [3], an *α* of 0.05, and a power of 80%; 20 participants were needed in each group. Because sex differences affect the anthropometric orofacial measures, minimally 40 participants were needed with almost equal numbers of females and males [11, 21, 32].

### Measuring the palatal surface area

**Fig. 1:**
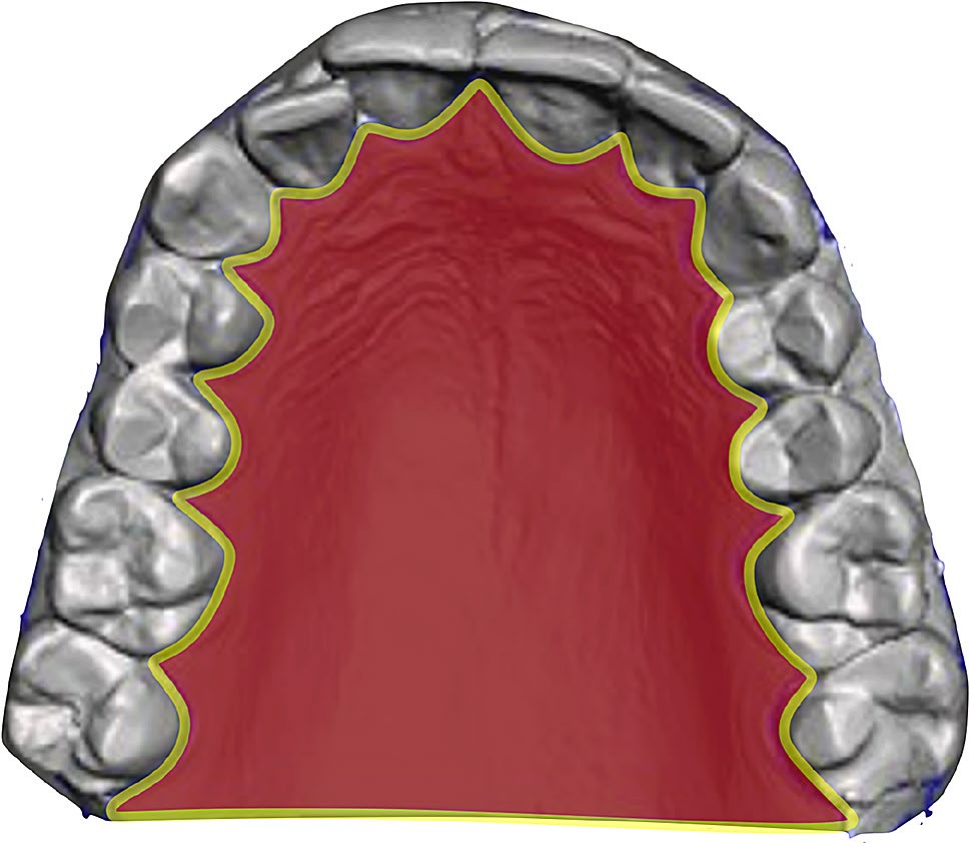
Schematic illustration of a typical example of palatal segmentation (in pink). The yellow line indicates the border of segmentation used for the palatal surface area (colour figure online)

To measure the palatal surface area, an intra-oral scan of the upper jaw including the palate (the whole hard palate and part of the soft palate) was taken with the TRIOS 3 scanner (3Shape, version 21.3.5, Copenhagen, Denmark). The scanning protocol of the manufacturer was followed when scanning the intra-oral upper jaw area. Scans were digitally saved in Polygon File Format (PLY) files. Subsequently, each PLY object was analysed twice in Meshmixer (Autodesk, San Rafael, CA, USA) by one researcher (ZA). This analysis involved manual separation of the palate by using the vibrating line including the visible fovea palatine as a cutoff for the length of the palate. Besides, all palatal mucosa including the gingiva around the upper teeth was included in the palatal surface (Fig. 1). After segmentation, the palatal surface areas (in mm^2^) were determined.

### Anthropometric measurements

Anthropometric measurements of the head and face were performed as described previously, using the same anthropometric landmarks (see Table 1) [3]. The distance between anthropometric landmarks was determined twice using an anatomical sliding caliper with electronic display showing distance in millimetres (mm). The two measurements were carried out by one researcher (ZA) on the same day.

**Table 1:** Definitions of anthropometric measurements in the present study

Anthropometric measurements	Anthropometric landmark
Length of the head	Vertex–gnathion
Width of the head	Straight line distance as measured with sliding caliper between the right external auditory meatus and left external auditory meatus
Depth of the head	Straight line distance as measured with a sliding caliper between the back of the head and glabella
Face height	Glabella–gnathion
Lower face height	Subnasale–gnathion
Nose height	Glabella–subnasale
Width of the mouth	Right chelion–left chelion
Upper face height	Glabella–lowest border of the upper lip
Upper lip height	Subnasale–lowest border of the upper lip
Mandible height	Gnathion–highest border of the lower lip
Mandibular length	Straight line distance as measured with a sliding caliper between the tragus and gnathion
Palatal width	Straight line distance from the central fissure of the upper right first molar (16) to the central fissure of the left first molar (26); if one or both teeth were extracted then the distance from the alveolar ridges of the estimated location of the first molars was used

### Statistical analysis

The data were processed in an electronic clinical data-management platform (CastorEdc, Castor, Amsterdam, the Netherlands) and then converted into SPSS, version 27.0 (IBM Corp SPSS Statistics, Armonk, NY, USA) for the statistical analysis. The Shapiro–Wilk test was used to assess the normality of the data. The data were presented as median, and their interquartile range (IQR), as most of the parameters were not normally distributed.

The intraclass correlation coefficient (ICC) was used to determine the degree of agreement between two palatal surface area measurements and between the two anthropometric measurements of the head and face. A two-way mixed, absolute agreement, average-measures ICC was calculated for these measurements [13, 19]. The ICC is indicative of poor (values less than 0.5), moderate (between 0.5 and 0.75), good (between 0.75 and 0.9) and excellent (greater than 0.90) reliability [17].

The mean of the two palatal surface area measurements and the various anthropometric measurements of the head and face were used for further analysis.

Female–male differences for the palatal surface area measurement and the anthropometric measurements of the head and face were explored with the Mann–Whitney *U* test.

The possible relations between the palatal surface area and anthropometric measurements were analysed with a bootstrapped Spearman rank correlation test (1000 × bootstrapping). The Spearman’s rho coefficient and bias-corrected accelerated (Bca) 95% confidence interval were extracted. Furthermore, the participants were stratified based on their sex. The size of the correlation coefficient was interpreted as poor (*r* = 0.1–0.2), fair (*r* = 0.3–0.5), moderate (*r* = 0.6–0.7) or very strong (*r* = 0.8–0.9) correlation [1]. All significance levels (*p*) were set at 0.05.

## Results

Fifty-one volunteers signed up for this study: 23 were female and 28 were male (45.1%: 54.9%). The average age was 42.6 ± 14.8 years (range 20–71 years). The average age of female and male participants did not differ significantly (Mann–Whitney *U* test *p* > 0.05).

### The palatal surface area and anthropometric measurements

The palatal surface area and the anthropometric measurements of the head and face are reported for the total study population as well as the female and male participants separately (Table 2). The median palatal surface area for the total study population was 2120.6 ± 1976.3–2232.0 mm^2^. The ICC for the palatal surface area measurements and the anthropometric measurements varied between 0.85 and 0.99, which was in the good or excellent range.

**Table 2:** The median and interquartile range (IQR) of anthropometric measurements (in mm) and the palatal surface area (mm^2^) for the total study population and stratified according to sex

Anthropometric measurements (mm)	ICC	Total (*N* = 51) Median ± IQR	Female (*N* = 23) Median ± IQR	Male (*N* = 28) Median ± IQR	*P* value female vs. male
Length of head	0.95	241.2 ± 230.1–245.6	230.1 ± 224.4–239.8	244.8 ± 240.1–249.6	< 0.001
Width of head	0.98	147.3 ± 141.9–154.3	141.9 ± 140.0–145.7	153.0 ± 147.1–158.2	< 0.001
Depth of head	0.99	198.4 ± 193.4–203.4	194.1 ± 189.9–198.3	201.3 ± 197.8–207.4	0.002
Face height	0.97	124.8 ± 115.0–129.7	115.0 ± 107.6–122.6	128.4 ± 124.9–131.6	< 0.001
Upper face height	0.98	79.7 ± 76.5–84.6	78.4 ± 75.5–80.1	83.1 ± 78.7–86.1	0.003
Lower face height	0.94	62.1 ± 57.0–68.1	57.2 ± 53.2–63.1	67.4 ± 60.3–69.4	0.001
Nose height	0.99	58.6 ± 54.6–61.5	57.6 ± 53.0–59.0	60.0 ± 56.2–63.1	0.016
Width of mouth	0.98	48.8 ± 45.4–53.2	48.3 ± 45.4–50.7	49.8 ± 45.7–54.0	0.219
Upper lip height	0.99	20.8 ± 19.6–23.9	20.2 ± 17.8–22.0	22.6 ± 20.6–25.2	0.004
Mandible height	0.98	41.5 ± 35.0–44.7	36.2 ± 32.7–41.7	43.6 ± 39.5–45.1	< 0.001
Mandibular length	0.85	144.2 ± 141.4–149.3	141.5 ± 133.2–144.7	144.3 ± 143.6–151.9	< 0.001
Palatal width	0.97	42.8 ± 40.0–45.7	42.4 ± 39.5–45.7	42.8 ± 41.3–46.0	0.374
*Surface area (mm*^*2*^*)*					
Palatal	0.96	2120.6 ± 1976.3–2232.0	2087.5 ± 1881.9–2184.2	2165.3 ± 2023.0–2257.3	0.069

*N* indicates the number of participants in each group. *p* value of Mann–Whitney *U* test is shown. ICC indicates the degree of agreement between the two independent measurements

Almost all anthropometric measurements showed significant differences between females and males (Mann–Whitney *U* test *p* < 0.05), where male participants showed higher values compared to females (Table 2). No sex-related differences were observed for the mouth and palatal widths. There was also no significant difference in the palatal surface area between females and males (Mann–Whitney *U* test *p* > 0.05).

### Relation between the palatal surface area and anthropometric measurements of the head and face

For the total study population, a significant correlation was found between the palatal surface area and the length of the head, the width of the head, face height, nose height, upper face height, upper lip height, mandibular length and palatal width (Table 3). The correlation coefficients for these correlations varied between 0.29 and 0.37, which indicates poor to fair correlations. These positive correlations indicate that larger dimensions of the head and face are associated with a larger palatal surface area. When the volunteers were stratified based on sex, the female palatal surface area correlated significantly with the mandibular length (0.46) and the palatal width (0.56) (Table 3). These correlations could be considered as fair.

**Table 3:** The correlations between the palatal surface area and anthropometric measurements for the total study population and stratified according to sex

Anthropometric measurements	Correlation coefficient with the palatal surface area		
	Total study population (*N* = 51)	Female participants (*N* = 23)	Male participants (*N* = 28)
Length of head	0.30 (− 0.01–0.58)^*^	NS (*p* = 0.13)	NS (*p* = 0.99)
Width of head	0.35 (0.06–0.61)^*^	NS (*p* = 0.09)	NS (*p* = 0.78)
Depth of head	NS (*p* = 0.27)	NS (*p* = 0.16)	NS (*p* = 0.44)
Face height	0.36 (0.14–0.56)^**^	NS (*p* = 0.60)	NS (*p* = 0.13)
Lower face height	NS (*p* = 0.19)	NS (*p* = 0.53)	NS (*p* = 0.46)
Nose height	0.31 (0.02–0.55)^*^	NS (*p* = 0.20)	NS (*p* = 0.24)
Width of mouth	NS (*p* = 0.17)	NS (*p* = 0.08)	NS (*p* = 0.96)
Upper face height	0.36 (0.10–0.58)^**^	NS (*p* = 0.18)	NS (*p* = 0.14)
Upper lip height	0.31 (0.02–0.56)^*^	NS (*p* = 0.20)	NS (*p* = 0.49)
Mandible height	NS (*p* = 0.37)	NS (*p* = 0.77)	NS (*p* = 0.22)
Mandibular length	0.29 (0.00–0.53)^*^	0.56 (0.20–0.78)^**^	NS (*p* = 0.37)
Palatal width	0.37 (0.10–0.63)^**^	0.46 (0.06–0.76)^*^	NS (*p* = 0.08)

*N* indicates the number of participants in each group. Data are expressed as the Spearman’s rho coefficient and bias-corrected accelerated (Bca) 95% confidence interval

*NS, *not significant, (*p* value of Spearman’s rho correlation)

^*^Spearman’s rho correlation coefficient *p* value <0.05

^**^Spearman’s rho correlation coefficient *p* value <0.01

## Discussion

This study aimed to assess the possible relation between the dimensions of the palatal surface area and anthropometric measurements of the head and face in living subjects. An intra-oral scanner was used to determine the palatal surface area. The excellent ICC for the palatal surface areas indicated the high reproducibility of the intra-oral scanner technique. Various head and face measurements had a significant correlation with the palatal surface area. When stratified by sex, significant correlations with the female palatal surface were found with the mandibular length and palatal width.

The adult palatal surface area was 2120.6 mm^2^, which was comparable to findings of other studies with a mean of 1990–2010 mm^2^ [3, 6, 15]. In these studies, the palatal surface areas were determined using foil impressions taken from stone models [6, 15], while another study used CBCT imaging and digital analysis [3]. Apparently, all methods used so far reveal comparable and representative results, as the reported palatal surface areas are in the same range. In addition, the technique presented in the current study, using an intra-oral scanner, adds up to this line of methods, as these had a very good reproducibility with an excellent ICC. Moreover, the intra-oral scanner has the beneficial effect of not using ionizing radiation and its technique is easy, safe and less laborious.

The palatal surface area in the current study did not differ between the two sexes. This finding is consistent with the results of two other studies [3, 6], while another study revealed that male participants had a significantly larger palatal surface areas compared to females [15]. This latter study, however, included females with a mean age 16.8 ± 8.02 years and males of 20.7 ± 13.4 years old [15]. These participants were considerably younger than the volunteers in the current study with a mean age of 42.6 ± 14.8 years. In this light, is has to be noted that maturation of female facial structures starts at an earlier age compared to males [24]. For this reason, in younger aged groups, there is a significant difference in palatal surface area between the two sexes, which explains why the study by Kerr et al*.* found significant differences in the palatal surface area measurements [15]. However, when investigating older subjects, such as the current study, these differences in the palatal surface area apparently disappeared.

In the current study, head and face proportions differed significantly between females and males. This finding is broadly supported by the work of other studies describing the effects of sex on anthropometric orofacial measures, mentioning larger measures for males when compared to females [11, 21, 32]. In our previous study, investigating cadavers with CBCT, comparable anthropometric differences between two sexes were observed [3]. In the cadaver study, the length of the head did not differ significantly in the two sexes, while in the current study there was a significant difference in the length of the head between the two sexes. This result could be explained by the limited number of cadavers used in the CBCT study (female *N* = 8, and male *N* = 5) [3] compared to the larger number of living subjects in the current study (female *N* = 23, and male *N* = 28).

In the current study, various anthropometric measurements had a significant correlation with the palatal surface area. This is in contrast with the CBCT study with human cadavers where only a statistically significant correlation between the length of the head and palatal surface area was observed. There are several possible explanations for this result. Firstly, the previous study included cadavers with possibly solidified soft tissues. Secondly, the number of included subjects might also influence this observation; the cadaver study had a possibly limited statistical power due to the limited number of cadavers used (*N* = 12). Although in the current study more significant correlations were found between palatal surface area and facial anthropometric measurements, most of these correlations were poor or fair (± 0.3). Finally, sex differences influenced these correlations, as males had significantly larger head and face proportions then females. For this reason, most of the significant correlations disappeared after stratifying the subjects based on their sex, especially for males. Females had a significant correlation between palatal surface with the mandibular length and the palatal width. Possibly, the face type of females attributed to this significant correlation. The face type of females is different compared to males; for females the most common face type is mesoprosop (medium-broad face) or euryprosop (short and wide), while for male it is the leptoprosop (long and narrow) and hyperleptoprosop [2, 31].

Previous studies measured not only the palatal surface, but also palatal volume. This palatal volume can contribute to explore the timing of surgery and surgical protocols [12, 20, 26]. In addition, palatal volume measurements can help to evaluate changes induced by treatment modalities such as rapid palatal expansion and in the orthopaedic treatment of cleft palate cases and to evaluate changes in orthodontic treatment [12, 20, 26]. Therefore, future studies exploring the relation between the palatal volume and anthropometric measurements are also warranted.

## Conclusion

An optical scanner was successfully used to determine the palatal surface area, as the ICC for the palatal surface area was in excellent range. Various head and face proportions had a significant correlation with the palatal surface area. When stratified by sex, significant correlations with the female palatal surface were found with the mandibular length and palatal width.

## Declarations

### Availability of data and materials

All data are available upon request from the corresponding author.
